# Water extract of *Spatholobus suberectus* inhibits osteoclast differentiation and bone resorption

**DOI:** 10.1186/1472-6882-13-112

**Published:** 2013-05-21

**Authors:** Hyunil Ha, Ki-Shuk Shim, Hyosun An, Taesoo Kim, Jin Yeul Ma

**Affiliations:** 1 KM-Based Herbal Drug Research Group, Korea Institute of Oriental Medicine, Daejeon, 305-811, Republic of Korea

**Keywords:** Osteoporosis, *Spatholobus suberectus*, RANKL, Osteoclasts, Bone resorption

## Abstract

**Background:**

Osteoclasts are primarily responsible for bone resorption. In many pathological bone diseases including osteoporosis and rheumatoid arthritis, osteoclasts are excessively activated. Thus, controlling of osteoclasts would be an effective therapeutic strategy for the treatment of excessive bone loss. The stem of *Spatholobus suberectus* has been widely used in traditional medicine to treat blood stasis syndrome and arthritis in Asia. In the present study, we investigated the effects and action mechanism of water extract of the stem of *Spatholobus suberectus* (WESS) on osteoclast differentiation and function.

**Methods:**

The effect of WESS on osteoclast differentiation was evaluated by counting tartrate resistant acid phosphatase-positive multinucleated cells in bone marrow-derived macrophages system and murine bone marrow cell-osteoblast coculture system. Bone resorption activity of mature osteoclast was examined on a calcium phosphate-coated plate. Actin ring structure of osteoclasts was detected fluorescently by staining for F-actin. Activation of signaling pathways and induction of transcription factors required for osteoclastogenesis were investigated by real-time PCR and Western blotting.

**Results:**

WESS effectively inhibited osteoclast differentiation from its precursors. The inhibitory effect of WESS on osteoclast differentiation was due to the suppression of osteoclastogenic transcription factors, c-Fos and nuclear factor of activated T cells cytoplasmic 1 expression, via preventing receptor activator of nuclear factor-κB ligand-induced early signaling pathways and decreasing c-Fos protein level in osteoclast precursors. Furthermore, WESS suppressed bone resorption activity of osteoclasts by disrupting actin ring structure.

**Conclusions:**

This study demonstrated that WESS inhibits osteoclast differentiation and function. These results suggest that WESS has a potential for treating pathological bone diseases caused by excessive bone resorption.

## Background

Bone remodeling is a coupled process consisting of osteoclastic bone resorption followed by osteoblastic bone formation throughout life [1]. It is necessary to repair damaged bone and to maintain mineral homeostasis. Progressive and excessive bone resorption by osteoclasts than bone formation causes an imbalance of bone remodeling which is characterized in several pathological bone diseases including osteoporosis, Paget’s disease, and rheumatoid arthritis [1].

Osteoclasts are exclusive bone-resorbing multinucleated cells formed by the proliferation, differentiation, and fusion of hematopoietic cells belonging to the monocyte/macrophage lineage [1,2]. When attached to bone matrix, multinucleated osteoclasts polarize their membrane to bone and secrete protons and lytic enzymes such as cathepsin K into the resorption lacuna surrounded by a tight sealing zone [1]. The sealing zone, a characteristic feature of functional osteoclast, isolates the resorptive microenvironment from the general extracellular space. It contains ring-like structures called actin ring consisting of F-actin dots [3].

Receptor activator of NF-κB ligand (RANKL) is the key cytokine that stimulates entire processes for the development of bone-resorbing osteoclasts: commitment, fusion, and activation [2,4]. RANKL is produced by several cell types including osteoblasts and mineralized matrix-embedded chondrocytes and osteocytes [2,5]. Upon binding to its receptor RANK on the cell surface of osteoclast precursors, RANKL induces the recruitment of adaptor molecules such as tumor necrosis factor receptor-associated factor 6, which activates multiple downstream signaling molecules including mitogen-activated protein kinases (MAPKs) and NF-κB. These signaling pathways in turn ultimately lead to induction and activation of transcription factors required for osteoclast differentiation and activation [6]. Those transcription factors include c-Fos and nuclear factor of activated T-cells cytoplasmic 1 (NFATc1). c-Fos, a component of activator protein-1, is induced by RANKL stimulation in osteoclast precursors, and mice lacking c-Fos are osteopetrotic due to impaired osteoclast development [7,8]. NFATc1 functions as a master transcription factor for osteoclast differentiation, and its induction is dependent on c-Fos [9,10].

There is a growing interest in the utilization of medicinal plants for prevention and treatment of bone disorders including osteoporosis [11]. To evaluate bone-protective potential of traditional Korean herbal medicines, we investigated the effects of water extracts of 150 herbal medicines on RANKL-induced osteoclast differentiation. Among them, the stem of *Spatholobus suberectus* showed relatively strong inhibitory activity against osteoclast differentiation without adversely affecting cell viability. In addition, the stem of *S. suberectus*, known as Ji Xue Teng in China and Gye-Hyeol-Deung in Korea, has been used to treat anemia, menstrual abnormalities, and arthritis in Asia [12]. It has been reported that the extracts of the stem of *S. suberectus* exhibit diverse biological functions including hematopoietic-supportive effects [13], anti-platelet effects [14], anti-inflammatory activities [15], and antioxidant activities [15,16], and anti-rheumatic effects [17,18]. However, to date the direct effects of *S. suberectus* on bone metabolism have not been studied. In the present study, we explored the anti-osteoclastogenic effect of water extract of the stem of *S. suberectus* (WESS) and its underlying molecular mechanism.

## Methods

### Reagents

The dried stem of *S. suberectus* was purchased from Yeongcheon herb (Yeongcheon, Korea). α-modified minimal essential medium (α-MEM), fetal bovine serum (FBS), BCA protein assay kit, and SuperSignal West Femto Maximum Sensitivity Substrate were purchased from Thermo Fisher Scientific Inc. (Rockford, IL, USA). Cell Counting Kit-8 was obtained from Dojindo Molecular Technologies Inc. (Tokyo, Japan). RNA-spin total RNA extraction kit, AccuPower RT-PreMix, and AccuPower GreenStar QPCR Master Mix were obtained from Bioneer (Daejeon, Korea). 1α,25-dihydroxyvitaminD3 (VitD_3_), *p*-nitrophenyl phosphate, and phalloidin-TRITC were purchased from Sigma-Aldrich (St. Louis, MO, USA). Antibodies specific for phospho-ERK1/2 (thr202/Tyr204), ERK, phospho-JNK1/2 (Thr183/Tyr185), JNK, phospho-p38 (Thr180/Tyr182), p38, phospho-p65 (Ser536), p65, phospho-IκBα (Ser32), and IκBα were from Cell Singling Technology (Danvers, MA, USA). Antibodies against c-Fos, NFATc1, β-actin, and GAPDH were from Santa Cruz Biotechnology (Santa Cruz, CA, USA). M-CSF and RANKL were kindly provided by Dr. Yongwon Choi (University of Pennsylvania School of Medicine).

### Preparation of WESS

A voucher specimen of *S. suberectus* (No. E188) was deposited in the herbal bank of KM-Based Herbal Drug Research Group, Korea Institute of Oriental Medicine. The dried stem of *S. suberectus* (50 g) was boiled for 3 h in 1 L of distilled water (DW). After filtration using testing sieves (150 μm) (Retsch, Haan, Germany), the extract was lyophilized and stored at 4°C before use. To prepare WESS, the lyophilized powder (yield: 7.35%) was re-suspended in distilled water, centrifuged at 10,000 × g for 5 min, and filtered through a 0.2 μm sterile filter.

### Animals

5-week-old male ICR mice (Orient Bio Inc., Seoul, Korea) were housed under constant environmental conditions (22 ± 1°C, 55 ± 10% humidity, and 12 h light/dark cycle) with free access to a standard animal diet and tap water. Bone marrow cells were collected from the tibias and femurs of male mice, after acclimatization for 1 week. Newborn ICR mice were purchased from Orient Bio Inc. for preparation of mouse calvarial osteoblasts. All animal procedures were performed according to the Guide for the Care and Use of Laboratory Animals of the National Institutes of Health. The experimental protocols were approved by the Institutional Animal Care and Use Committee at Korea Institute of Oriental Medicine (Reference number: 11-125 and 12-004).

### Cell culture and osteoclast differentiation

Bone marrow-derived macrophages (BMMs) were derived from mouse bone marrow cells and cultured in α-MEM complete medium containing 10% FBS and antibiotics (100 U/ml penicillin and 100 μg/ml streptomycin) in the presence of M-CSF (60 ng/ml) as described previously [19]. Cell viability of BMMs was determined using Cell Counting Kit 8, after 2 days of BMMs culture (1 × 10^4^ cells/well in a 96-well plate) with WESS and M-CSF (60 ng/ml). To differentiate BMMs into osteoclasts, BMMs (1 × 10^4^ cells/well) were cultured with M-CSF (60 ng/ml) and RANKL (100 ng/ml) for 4 days in 96-well plates. Mouse calvarial osteoblasts were obtained from calvariae of newborn ICR mice by enzymatic digestion as described previously [19]. For osteoclast differentiation from the coculture of osteoblasts and bone marrow cells, bone marrow cells (3 × 10^5^ cells/well) and osteoblasts (2 × 10^4^ cells/well) were cocultured with VitD_3_ (10 nM) in 48-well tissue culture plates for 6 days. All cultures were replenished with fresh medium on day 3.

For total tartrate-resistant acid phosphatase (TRAP) activity assay, cells were fixed in 10% neutral buffered formalin for 10 min, permeabilized with 0.1% Triton X-100 in PBS, and incubated with *p*-nitrophenyl phosphate as substrate according to the manufacturer’s instructions (Sigma-Aldrich) in a TRAP assay buffer (50 mM sodium tartrate and 0.12 M sodium acetate, pH 5.2). After 10 min of incubation at 37°C, the reaction was stopped with 1 M NaOH, and the absorbance was measured at 405 nm using a spectrophotometer. TRAP staining was carried out using Naphthol AS-MX phosphate and Fast Red Violet LB according to the protocol described in BD Biosciences Technical Bulletin #445. TRAP-positive multinucleated cells (TRAP(+)MNC) containing more than three nuclei were counted as osteoclasts.

### Retroviral gene transduction

Retrovirus packaging and BMM infection by using retroviral vectors pMX-IRES-green fluorescent protein (GFP) and pMX-constitutively active (CA)-NFATc1-IRES-GFP were performed as described previously [19]. BMMs infected with the retroviruses were further cultured in the presence of M-CSF (60 ng/ml) for 1 day and then treated as indicated. Ectopic expression of each construct was detected by a fluorescence microscope (Olympus IX53 inverted microscope), and cells were stained for TRAP.

### Real-time quantitative PCR (QPCR)

To evaluate mRNA levels of RANKL and OPG, osteoblasts (3 × 10^5^ cells/well in a 6-well plate) were pre-incubated with WESS for 3 h and stimulated with VitD_3_ for 24 h. To evaluate mRNA levels of NFATc1 and c-Fos in BMMs, BMMs (4 × 10^5^ cells/well in a 6-well plate) were pre-incubated with WESS for 3 h in the presence of M-CSF (60 ng/ml) and further cultured with RANKL (100 ng/ml) for indicated times. Total RNA was isolated with an RNA-spin total RNA Extraction Kit according to the manufacturer’s protocol. cDNA was synthesized from 1 μg of total RNA in AccuPower RT-PreMix according to the manufacturer’s protocol. SYBR green-based QPCR amplification was performed using cDNA diluted to 1:3, 10 pmol of primers, and AccuPower GreenStar QPCR Master Mix in the Applied Biosystems 7500 Real-Time PCR System (Applied Biosystems, Foster City, CA, USA). The PCR reaction consisted of 40 cycles of 94°C for 20 s and 60°C for 40 s. All reactions were run in triplicate, and data were analyzed using the 2^-ΔΔCT^ method. HPRT was used as an internal control to normalize RNA amount. The primer sequences used were described previously [19].

### Western blot analysis

BMMs treated as indicated were washed twice with ice-cold PBS and lysed in a protein extraction buffer containing 20 mM Tris-HCl, 150 mM NaCl, 1 mM EDTA, 1 mM EGTA, 1% NP-40, 0.1% SDS, and protease and phosphatase inhibitor cocktails (Roche Applied Science, Indianapolis, IN, USA) at 4°C. Total cell lysates were obtained by centrifugation at 10,000 × g for 15 min at 4°C. Protein concentration of lysates was determined with a BCA Protein Assay Kit. Protein samples (30 μg) were subjected to SDS-polyacrylamide gel electrophoresis and transferred to polyvinylidende fluoride membranes. Membranes were blocked with blocking buffer, 5% non-fat dry milk in TBST (10 mM Tris-HCl [pH 7.5], 150 mM NaCl, and 0.1% Tween 20), for 1 h at room temperature, probed with the indicated primary antibodies (1/1000 dilution) overnight at 4°C, and then washed with TBST three times for 10 min each. Afterward, membranes were incubated with horseradish peroxidase-conjugated secondary antibodies (1/4000 dilution) for 1 h at room temperature and washed with TBST three times. Chemiluminescent signals were detected on a LAS-4000 Luminescent Image Analyzer (Fuji Photo Film Co., Tokyo, Japan) with SuperSignal West Femto Maximum Sensitivity Substrate.

### Resorption pit assay

Mature osteoclasts were generated by coculturing of mouse bone marrow cells and osteoblasts with VitD_3_ (10 nM) on collagen gels for 6 days. The generated osteoclasts were replated on an Osteo Assay Surface plate (Corning, NY, USA), allowed to settle for 2 h, and then incubated with different concentrations of WESS for 24 h. After the incubation period, osteoclasts were visualized by TRAP staining. Resorption pits were photographed and analyzed by using Image J software, after removing cells by using sodium hypochlorite bleach.

### Actin ring formation assay

Osteoclasts were generated from BMM culture with M-CSF and RANKL on tissue culture plates as described above and then treated with different concentrations of WESS for 30 min. Cells were washed with PBS, fixed in 10% neutral buffered formalin for 10 min, permeabilized with 0.1% Triton X-100 in PBS for 5 min, and incubated with phalloidin-TRITC (0.2 μg/ml in PBS) for 15 min. After washing three times with PBS, F-actin stained with phalloidin-TRITC was photographed using a fluorescence microscope (Olympus IX53 inverted microscope).

### Statistical analysis

All quantitative data are presented as mean ± SD of three independent experiments. Statistical difference was determined by Student’s *t* test for two-group comparisons or by one-way analysis of variance followed by Tukey’s post-hoc test for multiple-group comparisons. A *p* value less than 0.05 were considered significant.

## Results

### Effect of WESS on osteoclast differentiation in bone marrow cell-osteoblast coculture

**Figure 1:**
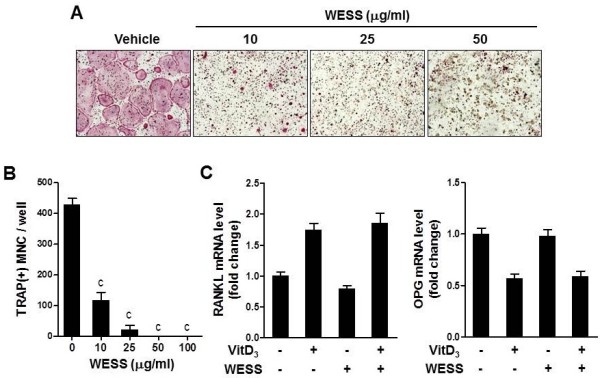
**Inhibitory effect of WESS on osteoclast differentiation in bone marrow cell-osteoblast coculture.** Mouse bone marrow cells and calvarial osteoblasts were cocultured with vehicle (DW) or WESS in the presence of VitD_3_ (10 nM) for 6 days. **(A)** Representative microscopic pictures of TRAP staining are shown at 100× magnification. **(B)** The number of TRAP-positive multinucleated osteoclasts (TRAP(+)MNC) containing three or more nuclei were counted. Data represents mean ± SD of three independent experiments. ^c^*P* < 0.001 vs. vehicle-treated control group. **(C)** Osteoblasts were pre-incubated with vehicle or WESS (25 μg/ml) for 3 h and stimulated with VitD_3_ (10 nM) for 24 h. RANKL and OPG mRNA levels were analyzed by QPCR.

It has been known that osteoblasts control osteoclast differentiation by expressing RANKL and its decoy receptor osteoprotegerin (OPG). Many osteoclastogenic factors such as VitD_3_, IL-1, and PTH stimulate osteoclast differentiation by increasing the ratio of RANKL to OPG [1]. To investigate whether WESS affects the ability of osteoblasts to support osteoclast differentiation, we tested in bone marrow cell-osteoblast coculture system. Treatment of the coculture with VitD_3_ for 6 days induced TRAP-positive multinucleated osteoclasts, which was dramatically inhibited by WESS in a dose-dependent manner (Figure 1A and B). We next examined whether WESS could regulate the expression of RANKL and OPG in osteoblasts. VitD_3_ increased RANKL mRNA levels and decreased OPG mRNA levels. Pretreatment with 25 μg/ml of WESS, a sufficient concentration to inhibit osteoclast differentiation, did not affect RANKL and OPG mRNA expression in basal or VitD_3_-stimulated osteoblasts (Figure 1C).

### Effect of WESS on RANKL-induced osteoclast differentiation

**Figure 2:**
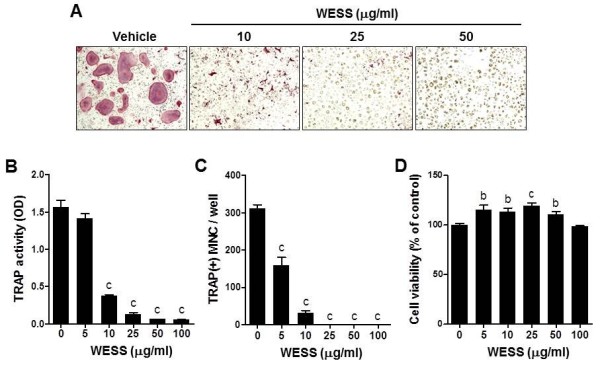
**Inhibitory effect of WESS on osteoclast differentiation in BMMs.** BMMs were cultured with vehicle or WESS in the presence of M-CSF (60 ng/ml) and RANKL (100 ng/ml) for 4 days. **(A)** Representative microscopic pictures of TRAP staining. **(B)** Total cellular TRAP activity. **(C)** The number of TRAP(+)MNC containing three or more nuclei. **(D)** Effect of WESS on the viability of BMMs in the presence of M-CSF (60 ng/ml). All bar graphs represent mean ± SD of three independent experiments. ^b^*P* < 0.01, ^c^*P* < 0.001 vs. control group.

Because the inhibitory effect of WESS on osteoclast formation in coculture was not due to changes in RANKL and OPG expression in osteoblasts, we next investigated the possibility that WESS inhibits osteoclast differentiation via directly acting on osteoclast precursor cells. When BMMs (osteoclast precursors) were cultured with RANKL in the presence of M-CSF for 4 days, numerous TRAP-positive multinucleated osteoclasts were generated. WESS inhibited RANKL-induced TRAP activity and osteoclast formation in a dose-dependent manner (Figure 2A-C). Consistent with the results of coculture experiment, a strong inhibition was observed at 25 μg/ml concentration of WESS. The inhibitory effect of WESS was not due to cellular toxicity. Rather, it increased cell viability of BMMs at concentrations of 5-50 μg/ml. Thus, 25 μg/ml of WESS was chosen for the subsequent experiments to investigate the anti-osteoclastogenic effect of WESS.

**Figure 3:**
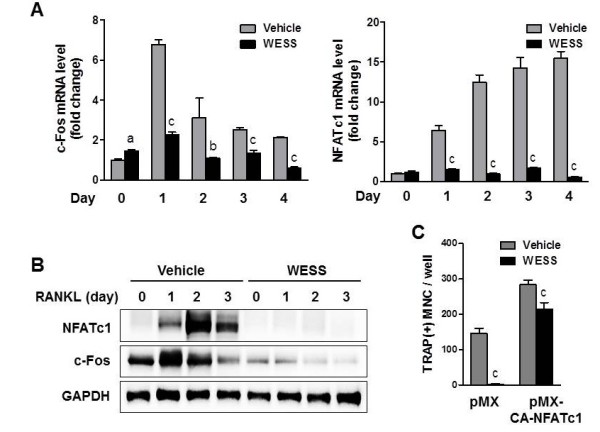
**Inhibitory effect of WESS on RANKL-induced c-Fos and NFATc1 expression in BMMs.** BMMs were pre-incubated with vehicle or WESS (25 μg/ml) for 3 h and further cultured in the presence of M-CSF (60 ng/ml) and RANKL (100 ng/ml) for 4 days. Total RNA and cell lysates were obtained at the indicated days. **(A)** c-Fos and NFATc1 mRNA levels were analyzed by QPCR. ^a^*P* < 0.05, ^b^*P* < 0.01, ^c^*P* < 0.001 vs. vehicle-treated control group. **(B)** Total cell lysates were subjected to Western blot analysis with antibodies specific for c-Fos and NFATc1. GAPDH was used as a loading control. **(C)** BMMs infected with retroviruses expressing either pMX-IRES-GFP (pMX) or pMX-CA-NFATc1-IRES-GFP (pMX-CA-NFATc1) were cultured with vehicle or WESS (25 μg/ml) in the presence of M-CSF (60 ng/ml) and RANKL (100 ng/ml) for 4 days. The number of TRAP(+)MNC containing more than three nuclei with GFP expression were counted. Data represents mean ± SD of three independent experiments. ^c^*P* < 0.001 vs. vehicle-treated control group.

### Effect of WESS on RANKL-induced c-Fos and NFATc1 expression

To address the inhibitory mechanism of WESS on RANKL-induced osteoclastogenesis, we explored the effect of WESS on the expression of c-Fos and NFATc1, key transcription factors for osteoclast differentiation [8,9]. Stimulation of BMMs with RANKL increased c-Fos mRNA and protein levels reaching a peak at day 1, followed by NFATc1 induction. In the absence of RANKL, WESS dramatically decreased c-Fos protein but not mRNA level. In addition, WESS abrogated RANKL-induced c-Fos and NFATc1 induction (Figure 3A and B). The inhibitory effect of WESS on osteoclast differentiation was overcome by ectopic expression of CA-NFATc1 in BMMs (Figure 3C).

### Effect of WESS on RANKL-induced early signaling pathways

**Figure 4:**
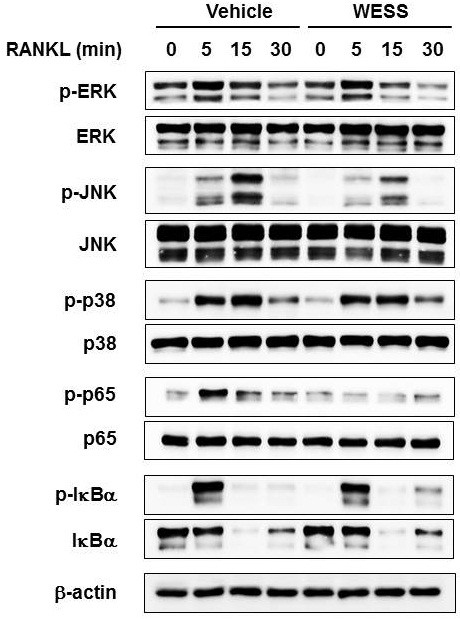
**Inhibitory effect of WESS on RANKL-induced activation of JNK and p65 in BMMs.** BMMs were pre-treated with vehicle or WESS (25 μg/ml) for 3 h and stimulated with RANKL (100 ng/ml). Total cell lysates were prepared at the indicated time points and then subjected to Western blot analysis using the indicated antibodies. β-actin was used as a loading control.

To gain more insights into the inhibitory mechanism of WESS on RANKL-induced osteoclastogenesis, we explored the effect of WESS on RANKL-induced activation of MAPKs and NF-κB. These signaling pathways are involved in RANKL-induced c-Fos expression and osteoclast differentiation [7,20-22]. WESS suppressed RANKL-induced activation of JNK but not ERK and p38 MAPKs, assessed by Western blotting with phosphorylated form-specific antibodies (Figure 4). It also abrogated RANKL-induced p65 phosphorylation (Ser536) without affecting IκBα phosphorylation and degradation (Figure 4).

### Effect of WESS on bone resorption activity

**Figure 5:**
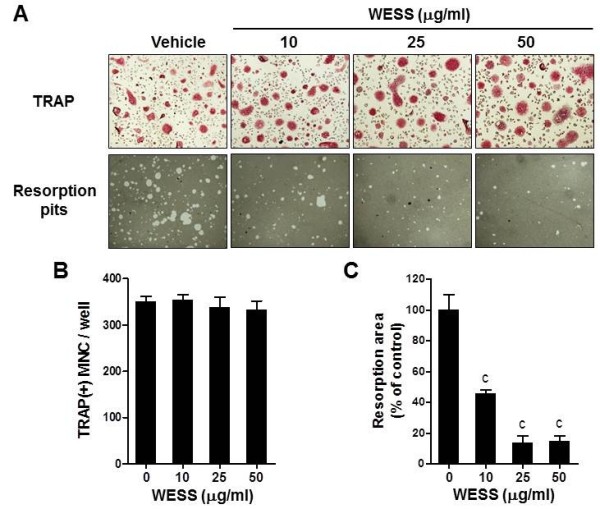
**Inhibitory effect of WESS on bone resorption activity of mature osteoclasts.** Mature osteoclasts generated on collagen gels were replated on an Osteo Assay Surface plate and cultured with vehicle or WESS for 24 h. **(A)** Representative microscopic pictures of TRAP staining (upper panel) and resorption pits (down panel). **(B)** The number of TRAP(+)MNC containing three or more nuclei. **(C)** Relative resorption area. Data represents mean ± SD of three independent experiments. ^c^*P* < 0.001 vs. vehicle-treated controls.

**Figure 6:**
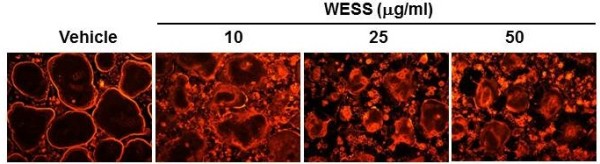
**Inhibitory effect of WESS on actin ring structure of mature osteoclasts.** Mature osteoclasts were treated with vehicle or WESS for 30 min. Osteoclasts were fixed, and F-actin was visualized by fluorescence microscope with phalloidin-TRITC staining.

Bone resorption is the unique function of osteoclasts. To investigate whether WESS affects bone resorption activity of osteoclasts, mature osteoclasts were cultured on a plate coated with an inorganic crystalline calcium phosphate designed to mimic bone mineral. After 24 h of culture, numerous resorption pits by osteoclasts were observed on the plate in vehicle-treated control group. WESS dose-dependently reduced bone resorption area (Figure 5A and C) without affecting the number of osteoclasts (Figure 5A and B), indicating that WESS inhibits bone resorption activity of osteoclasts. The bone resorption function of osteoclasts depends on dynamic regulation of the actin cytoskeleton. Actin ring structure is a characteristic cytoskeletal feature of functional osteoclasts [3]. Therefore, we next examined whether WESS affects actin ring structure of mature osteoclasts. In mature osteoclasts on tissue culture plates, F-actin was arranged into a ring-like structure (actin ring) at the cell periphery. Treatment of mature osteoclasts with WESS caused both shrinkage of osteoclasts and disruption of actin ring structure in a dose-dependent manner (Figure 6).

## Discussion

The stem of *S. suberectus* has been used for the treatment of inflammation and arthritis in Asia. Although WESS was shown to exhibit beneficial effects on collagen-induced arthritis by its immunomodulatory effects [18], its direct action on bone metabolism remains unknown. Here we have demonstrated for the first time that WESS inhibits osteoclast differentiation and bone resorption function.

WESS inhibited VitD_3_-induced osteoclast differentiation in the coculture system without affecting VitD_3_-induced changes in RANKL and OPG expression in osteoblasts. It also inhibited RANKL-induced osteoclast differentiation in BMM culture with similar potency. We found that WESS inhibits RANKL-induced c-Fos and NFATc1 induction in BMMs, and ectopic expression of CA-NFATc1 in BMMs reverses the inhibitory effect of WESS on osteoclast differentiation. These results suggest that the anti-osteoclastogenic effect of WESS is mainly caused by suppressing RANK signaling to induce c-Fos and NFATc1 expression in osteoclast precursors.

In studies on mechanisms underlying the inhibitory action of WESS, we found that WESS inhibits RANKL-induced JNK activation without affecting ERK and p38 activation in osteoclast precursors. Previous experiments using interferences of MKK7, JNK, and c-Jun with dominant-negative and small interfering RNA knockdown approaches have demonstrated that the sequential activation of the MKK7-JNK-c-Jun signaling pathway mediates RANKL-induced NFATc1 expression and osteoclastogenesis [23]. In addition, treatment with SP600125, a specific JNK inhibitor, also impaired c-Fos and NFATc1 expression as well as osteoclastogenesis in response to RANKL [22]. Thus, these observations suggest that WESS interferes with c-Fos and NFATc1 induction at least in part by inhibiting RANKL-induced JNK activation, thereby abrogating osteoclastogenesis.

Genetic studies have demonstrated that NF-κB signaling pathway plays a crucial role in osteoclastogenesis [6]. It has been suggested that NF-κB functions upstream of c-Fos during RANKL-induced osteoclastogenesis [7]. The classic NF-κB signaling pathway involves activation of the IκB kinase (IKK) complex that leads phosphorylation and degradation of IκBα, allowing nuclear translocation of NF-κB complexes containing the p50 and p65 subunits. The transcriptional activity of NF-κB can be controlled by various post-translational modifications, including p65 phosphorylation and acetylation [24]. In the present study, WESS did not affect RANKL-induced IκBα phosphorylation and degradation. However, WESS abrogated RANKL-induced p65 phosphorylation on Ser536. The phosphorylation of p65 on Ser536 in the transactivation domain has been shown to promote NF-κB transcriptional activity in response to inflammatory stimuli [25-27]. In addition, it has been reported that transforming growth factor-beta-activated kinase 1-MKK6-p38 signaling pathway participates to RANKL-induced NF-κB activation and osteoclastogenesis by stimulating p65 phosphorylation on Ser536 [28]. Therefore, our study suggests that the inhibitory effect of WESS on NF-κB activation may contribute to its anti-osteoclastogenic action. Given that IKKβ mediates RANKL-induced IκBα phosphorylation and degradation in osteoclast precursors [29], our findings suggest that WESS might target IKKβ-independent Ser536 protein kinases that include IKKα, IKKϵ, NF-κB activating kinase, bruton’s tyrosine kinase, protein kinase D3, and p38 [25-28,30].

In addition to inhibition of RANKL-induced c-Fos mRNA and protein expression, WESS dramatically decreased c-Fos protein level without negatively affecting c-Fos mRNA level in the absence of RANKL. IFN-β has been shown to inhibit c-Fos protein synthesis via activation of IFN-stimulated transcriptional factor 3 in osteoclast precursors [31]. However, the molecular mechanisms involved in the reduction of c-Fos protein expression by WESS remain to be elucidated.

Besides inhibiting osteoclast differentiation, WESS decreased bone resorption activity of mature osteoclasts in a dose-dependent manner, which was accompanied by a rapid disruption of actin ring structure in mature osteoclasts. Antioxidants such as N-acetyl-L-cysteine and glutathione were shown to inhibit actin ring formation and bone resorption activity of osteoclasts [32]. In contrast, reactive oxygen species showed the opposite effects [32,33]. Notably, WESS was found to exhibit the highest antioxidant activity among 45 Chinese herbs that regulate blood circulation [16]. Given the pivotal role of actin ring structure in bone resorption activity of osteoclasts [3], these observations collectively suggest that the strong antioxidant activity of WESS might contribute to its anti-resorptive activity by disrupting actin ring structure.

Chemical analyses have found that ethanol and methanol extracts of the dried stems of *S. suberectus* contain several flavonoids including genistein, daidzein, and isoliquiritigenin [34,35]. Although there is a lack of information on chemical compounds contained in WESS, WESS was shown to have high contents of total phenolics (24.11 GAE mg/g) and total flavonoids (165.16 QE mg/g) [15]. Flavonoids possess powerful antioxidant properties due to their polyphenolic chemical structure [36]. In addition, some flavonoids such as genistein, daidzein, and epigallocatechin-3-gallate have been shown to inhibit osteoclastogenesis by suppressing c-Fos expression [22,37]. Therefore, it is likely that certain components of WESS, possibly flavonoids, exert potent inhibitory effects on osteoclast differentiation and function by working together rather than separately.

## Conclusions

We have demonstrated that WESS suppresses osteoclast differentiation by inhibiting RANKL-induced signaling pathways and decreasing c-Fos protein level and also attenuates bone resorption activity of mature osteoclasts by disrupting actin ring structure. Our results provide scientific evidence to support the traditional use of *S. suberectus* to treat arthritis and suggest that WESS is potentially useful in treating other bone destructive diseases caused by excessive bone resorption.

## Competing interests

All authors declared that they have no competing interests.

## Authors’ contributions

HH, TK, and JYM participated in the design of the study and manuscript preparation. HH, KSS, and HA carried out the experiments and analyzed the data. All authors read and approved the final manuscript.

## Pre-publication history

The pre-publication history for this paper can be accessed here:

http://www.biomedcentral.com/1472-6882/13/112/prepub
